# Clinical Course and Electron Microscopic Findings in Lymphocytes of Patients with *DRAM2*-Associated Retinopathy

**DOI:** 10.3390/ijms21041331

**Published:** 2020-02-16

**Authors:** Kazuki Kuniyoshi, Takaaki Hayashi, Shuhei Kameya, Satoshi Katagiri, Kei Mizobuchi, Toshiaki Tachibana, Daiki Kubota, Hiroyuki Sakuramoto, Kazushige Tsunoda, Kaoru Fujinami, Kazutoshi Yoshitake, Takeshi Iwata, Tadashi Nakano, Shunji Kusaka

**Affiliations:** 1 Department of Ophthalmology, Kindai University Faculty of Medicine, Osaka 589-8511, Japan; sakurahiro0623@yahoo.co.jp (H.S.); kusaka-ns@umin.net (S.K.); 2 Department of Ophthalmology, The Jikei University School of Medicine, Tokyo 105-8461, Japan; takaaki@amy.hi-ho.ne.jp (T.H.); ktgr_two_ai@icloud.com (S.K.); kei10151202@icloud.com (K.M.); tnakano@jikei.ac.jp (T.N.); 3 Department of Ophthalmology, Katsushika Medical Center, The Jikei University School of Medicine, Tokyo 125-8506, Japan; 4 Department of Ophthalmology, Nippon Medical School Chiba Hokusoh Hospital, Chiba 270-1694, Japan; shuheik@nms.ac.jp (S.K.); 4d.freewill27@gmail.com (D.K.); 5 Core Research Facilities for Basic Science, Research Center for Medical Sciences, The Jikei University School of Medicine, Tokyo 105-8461, Japan; t-tachibana@jikei.ac.jp; 6 Sakuramoto Eye Clinic, Osaka 586-0001, Japan; 7 Laboratory of Visual Physiology, Division of Vision Research, National Institute of Sensory Organs, National Hospital Organization Tokyo Medical Center, Tokyo 152-8902, Japan; tsunodakazushige@kankakuki.go.jp (K.T.); k.fujinami@ucl.ac.uk (K.F.); 8 Division of Molecular and Cellular Biology, National Institute of Sensory Organs, National Hospital Organization Tokyo Medical Center, Tokyo 152-8902, Japan; akyoshita@g.ecc.u-tokyo.ac.jp (K.Y.); takeshi.iwata@kankakuki.go.jp (T.I.); 9 Graduate School of Agricultural and Life Sciences, Faculty of Agriculture, The University of Tokyo, Tokyo 113-8657, Japan

**Keywords:** retinitis pigmentosa, *DRAM2*, inherited retinal dystrophy, macular degeneration, electroretinogram, rod-cone dystrophy, visual field, electron microscopy, lymphocytes, autophagy

## Abstract

*DRAM2*-associated retinopathy is a rare inherited retinal dystrophy, and its outcome has not been determined. A single retinal involvement by a mutation of the *DRAM2* gene is unexplained. We found three unrelated patients with a disease-causing *DRAM2* variant in a biallelic state from 1555 Japanese individuals of 1314 families with inherited retinal dystrophy. We reviewed their medical records and examined their peripheral lymphocytes by transmission electron microscopy (TEM). *Patient 1* was a 38-year-old woman who complained of night blindness and reduced vision. She developed macular degeneration at age 43 years. *Patients 2* and *3* were a man and a woman both of whom noticed night blindness in their 30s. Both had a degeneration in the macula and midperiphery in their 40s, which progressed to a diffuse retinal degeneration in their 60s when their vision was reduced to hand motions. Three novel *DRAM2* variants were identified. TEM of the lymphocytes of *Patients 1* and *2* showed abnormal structures in 40.6% and 0.3% of the peripheral lymphocytes, respectively. We concluded that the *DRAM2*-associated retinopathy of our patients was a progressive rod-cone dystrophy, and the visual outcome was poor. The systemic effect of *DRAM2* mutations may be compensable and have variations.

## 1. Introduction

DNA-damage regulated autophagy modulator 2 (*DRAM2*; MIM #613360; GenBank NM_178454.4) is also known as transmembrane protein 77 (TMEM77). The *DRAM2* gene is located at locus 13.3 on the short arm of chromosome 1 and encodes a 266-amino acid transmembrane protein, which plays a role in autophagy induction [1,2] and tumor suppression [3]. *DRAM2* is expressed in various tissues, including the placenta, heart, spleen, and lymph nodes [1,4].

In the retina, *DRAM2* is located in lysosomes, the inner segments of the photoreceptors, and the apical surface of the retinal pigment epithelial (RPE) cells [5]. Mutations of *DRAM2* can cause cancer and neurodegeneration. An autosomal recessive cone-rod dystrophy with macular involvement is reported as *DRAM2*-associated retinopathy (CORD21) [5,6,7,8]. It is a rare inherited retinal dystrophy, and only 21 patients in 10 families have been reported [5,6,7,8]. The initial characteristic of retinopathy is macular degeneration, which is usually detected in the third or fourth decade of life. The macular degeneration is followed by retinal and RPE degeneration in the midperiphery. The findings at the advanced stage of *DRAM2*-associated retinopathy have not been reported, and a single retinal involvement by a mutation of the *DRAM2* gene is unexplained.

This study aimed to determine the clinical course of three patients with *DRAM2*-associated retinopathy at an advanced stage of the retinopathy and to present the effects of the *DRAM2* variants on the peripheral lymphocytes, determined by transmission electron microscopy (TEM).

## 2. Results

**Figure 1:**
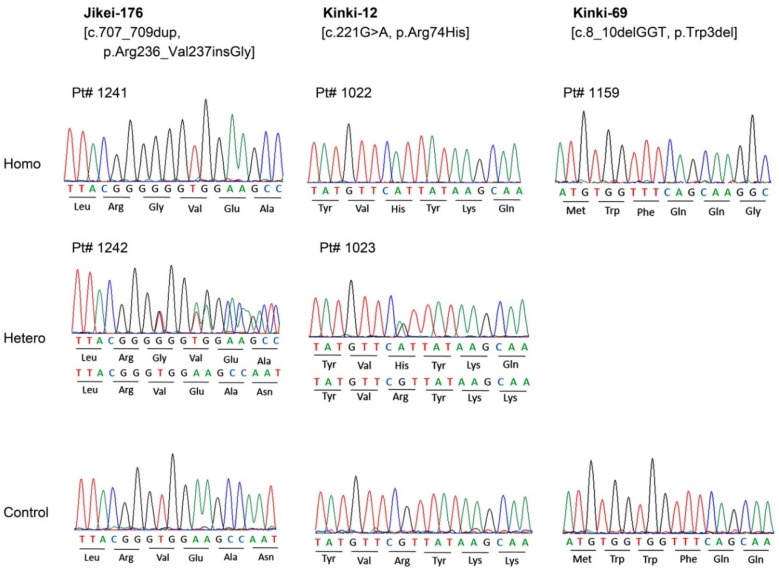
Results of Sanger sequencing; sequence chromatograms of identified *DRAM2* variants.

Initially, the whole exome sequencing (WES) data of 1555 patients of 1314 Japanese families with inherited retinal dystrophy were examined. A man and two women of three unrelated families were found to have disease-causing *DRAM2* variants. Thus, *DRAM2* made up 0.2% of the inherited retinal dystrophies. The results of Sanger sequences on the patients and their family members are shown in Figure 1, and their *DRAM2* variants data are summarized in Tables S1 and S2.

**Figure 2:**
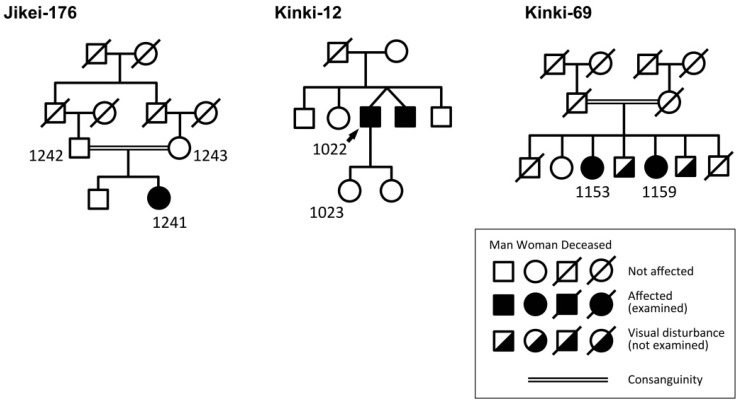
Pedigrees for the segregation analysis.

**Figure 3:**
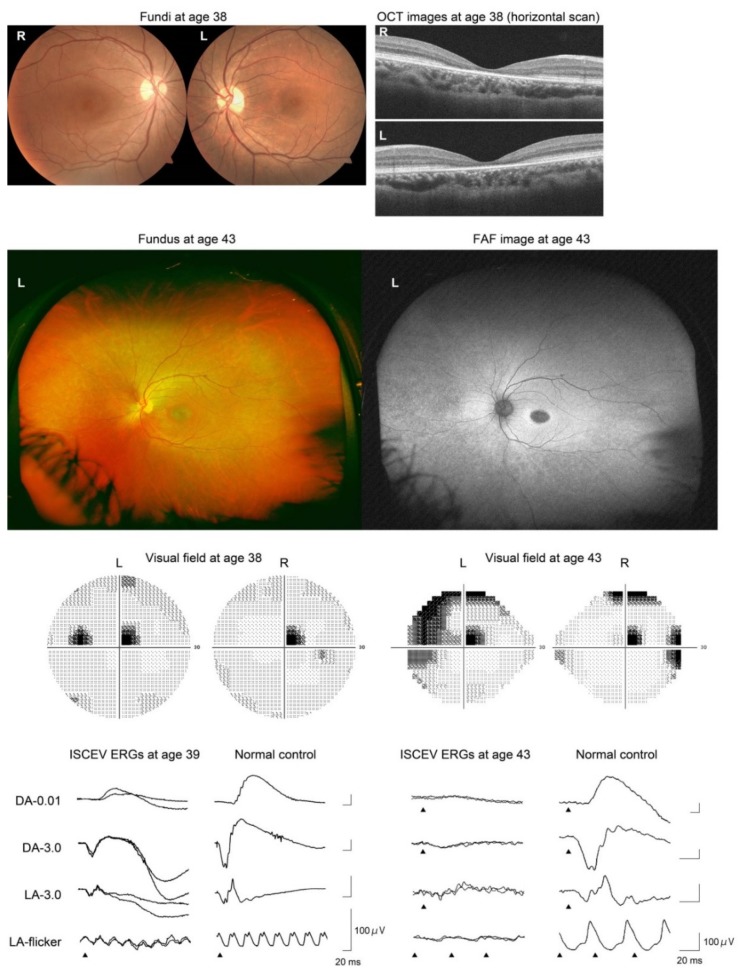
Results of fundus photography, fundus autofluorescence (FAF) imaging, optical coherence tomographic (OCT) imaging, Humphrey static visual field testing, and International Society for Clinical Electrophysiology of Vision (ISCEV)-standard full-field electroretinography (ERG) in *Patient 1* (Jikei-176-1241). The fundus and FAF images were obtained by an ultra-wide-field fundus camera (Optos) at age 43 years. This patient had a homozygous variant, c.707_709dup, p.Arg236_Val237insGly, in the *DRAM2* gene.

**Figure 4:**
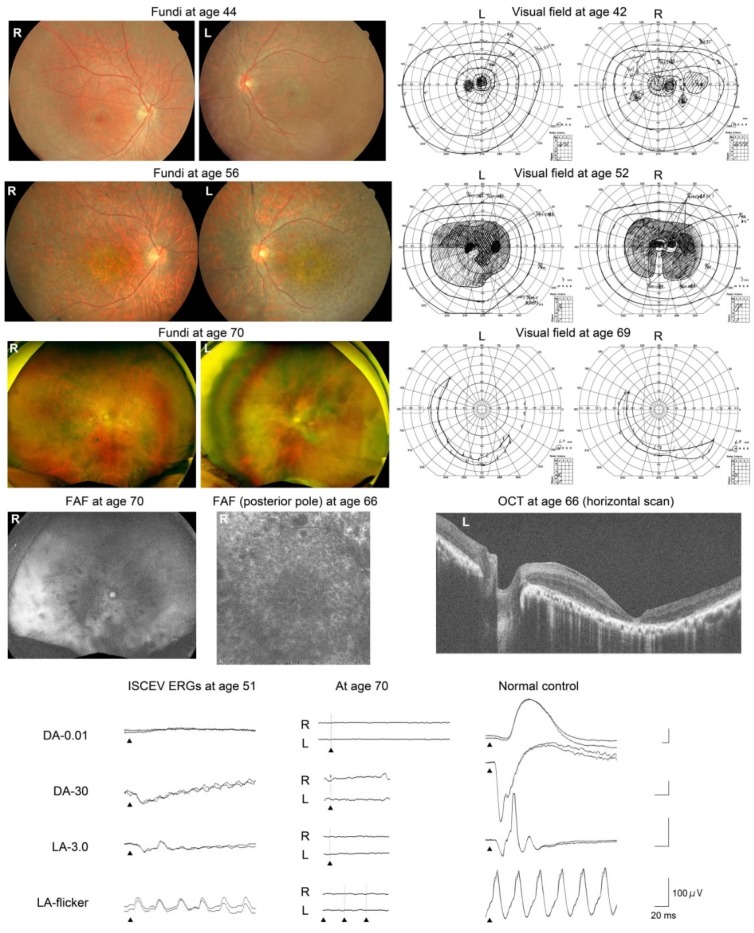
Results of fundus photography, FAF imaging, Goldmann kinetic visual field testing, OCT imaging, and ISCEV ERG in *Patient 2* (Kinki-12-1022). This patient had a homozygous variant, c.221G>A, p.Arg74His, in the *DRAM2* gene.

**Figure 5:**
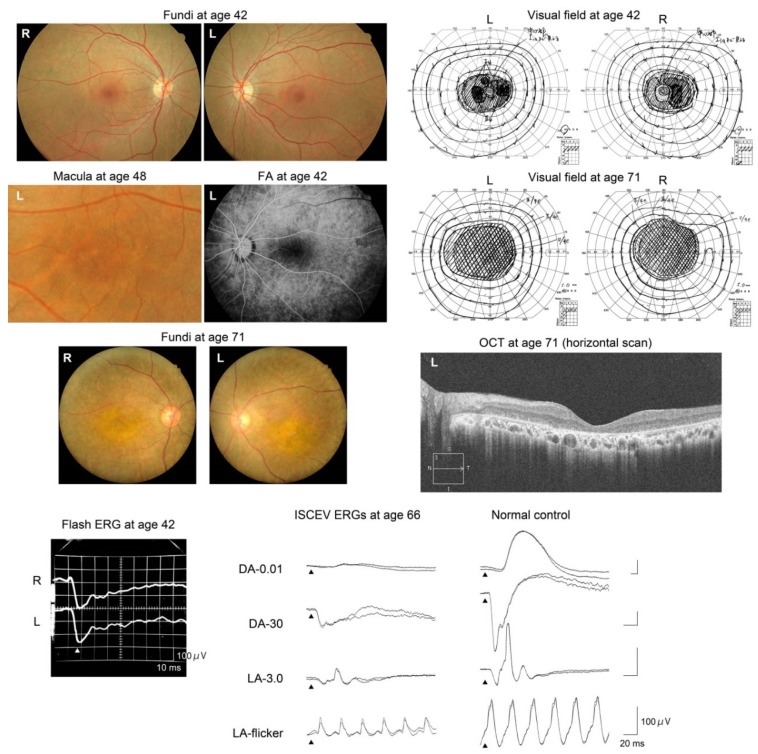
Results of fundus photography, fluorescein fundus angiography (FA), Goldmann kinetic visual field testing, OCT imaging, and ISCEV ERGs in *Patient 3* (Kinki-69-1159). This patient had both homozygous *DRAM2* variants, c.8_10delGGT, p.Trp3del and a heterozygous *EYS* variant, p.Gly843Glu.

**Figure 6:**
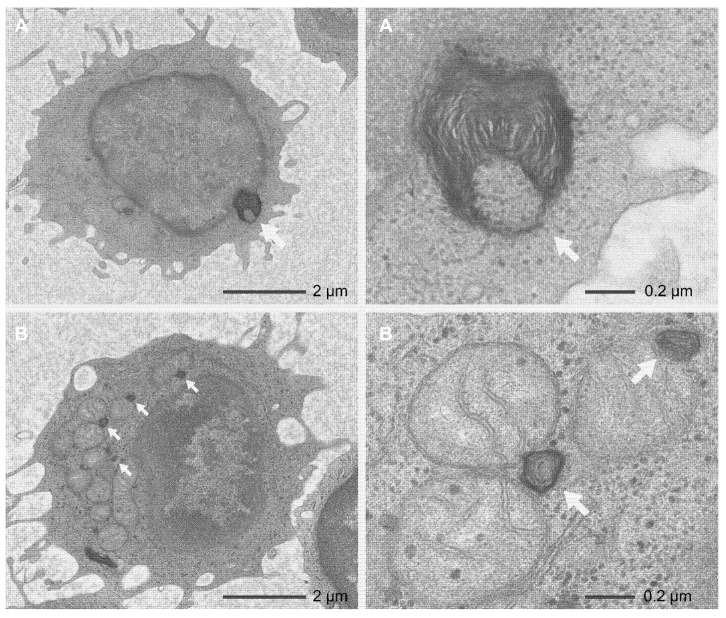
Transmission electron microscopic images of the peripheral lymphocytes in *Patient 1*. The left column shows the entire images of lymphocytes, and the right column shows the magnified images focusing on myelin-like lamellar structures. The images show myelin-like lamellar structures (arrows) around the nucleus (**A**) and mitochondria (**B**).

Pedigrees of the patients are shown in Figure 2, and their clinical courses are shown in Table 1 and Figure 3, Figure 4 and Figure 5. The results of the TEM are shown in Figure 6.

### 2.1. Genetic Studies

No other variants of retinal disease-associated genes were detected in these 1314 families except for *DRAM2* and *EYS* variants with high allele frequency (HAF) in a biallelic state.

### 2.2. DRAM2 Variants

Three homozygous *DRAM2* variants were identified by WES with target analysis of retinal disease-associated genes, viz., c.707_709dup, p.Arg236_Val237insGly in one family (Jikei-176); c.221G>A, p.Arg74His in one family (Kinki-12); and c.8_10delGGT, p.Trp3del in one family (Kinki-69) (Figure 1, Tables S1 and S2). These three variants have not been reported as disease-causing. Two families, Jikei-176 and Kinki-69, had histories of consanguineous marriages (Figure 2).

### 2.3. In Silico Molecular Genetic Analysis

The detailed results of *in silico* molecular genetic analysis for the three detected *DRAM2* variants are presented in Tables S1 and S2. The allelic frequencies for the three *DRAM2* variants in the general population of Total (gnomAD [9])/East Asian (gnomAD [9])/Japanese (HGVD [10]) were 0.000%/0.000%/0.000% for the p.Arg236_Val237insGly variant, 0.001%/0.000%/0.000% for the p.Arg74His variant and 0.012%/0.025%/0.061% for the p.Trp3del variant, respectively.

The functional prediction was assessed for the three variants. The pathogenicity classification according to the ACMG guideline [11] was “Likely Pathogenic” for the p.Arg236_Val237insGly and p.Arg74His variants, and “Uncertain” significance for the p.Trp3del variant.

### 2.4. EYS Variant

An *EYS* variant was also found in the Kinki-69 family (Table S3). *Patient 3* (1159) from the family had a heterozygous variant of *EYS* (p.Gly843Glu) with HAF. Although the allelic frequency of this variant was relatively high in the Japanese population, it was considered as potentially pathogenic only in the biallelic state [12,13,14,15]. Her older sister (1153 in Figure 2), who had similar and more severe retinopathy than that in *Patient 3* (1159), had a homozygous *EYS* variant (p.Gly843Glu) in addition to the homozygous *DRAM2* variant (p.Trp3del). *Patient 1153* was excluded from this report because her retinopathy was possibly affected by both the *DRAM2* and *EYS* variants. The clinical course of *Patient 1153* is presented in Figure S1.

### 2.5. Clinical Course of Patients

The visual acuities and refractive errors of the patients are shown in Table 1.

*Patient 1* (Jikei-176-1241, Figure 3) was a generally healthy 38-year-old Japanese woman. Her parents were first cousins, as shown in Figure 2. She noticed a difficulty in night vision at age 19 years and reduced visual acuity at age 37 years. Initial funduscopy at age 38 years showed fine white dots in the macula, and granular macular degeneration appeared 5 years later, at age 43 years. Fundus autofluorescence (FAF) imaging at age 38 years showed abnormal hypo-autofluorescence surrounded by a ring-shaped hyper-autofluorescence in the macula. Five years later, additional abnormal hypoautofluorescence areas appeared in the midperiphery. The optical coherence tomographic (OCT) images showed a disrupted ellipsoid zone and thinning of the outer retinal layers at the macula. Visual field tests revealed a central scotoma at age 38 years, and peripheral field defects appeared at age 43 years. The full-field electroretinograms (ERGs) were subnormal for both rod and cone responses at age 39 years, and they were almost nonrecordable 4 years later at age 43 years (Figure 3).

*Patient 2* (Kinki-12-1022, Figure 4) was a generally healthy 42-year-old Japanese man. His parents were not consanguineous, but they grew up in the same village. His brother, who was an identical twin, was diagnosed with retinal dystrophy in another hospital at age 40 years (Figure 2). The patient noticed a reduction of his visual acuity when he was 36 years old and night blindness at age 37 years. At the initial visit, when he was 42 years old, his fundi showed macular and midperipheral RPE degenerations in both eyes. No retinal pigmentation was seen. The degenerations gradually progressed to a diffuse retinal degeneration with vessel attenuation and bone-spicule pigmentation in his seventh decade of life. Goldmann kinetic perimetry revealed a central scotoma in both eyes. In addition, a large area surrounding the central scotoma had reduced sensitivity. During the follow-up period, he developed a large central scotoma, and his vision finally deteriorated to hand motions at age 70 years (Table 1). FAF images showed a mosaic pattern with hyper- and hypo-autofluorescence. OCT images revealed atrophy of the outer retinal layers. The full-field rod ERGs were nonrecordable, and cone ERGs were reduced. He was diagnosed with rod-cone dystrophy at age 51 years. The ERGs were extinguished at age 70 years (Figure 4).

*Patient 3* (Kinki-69-1159, Figure 5) was a 42-year-old Japanese woman when she first visited our clinic. She reported that she had had difficulty seeing in dark environments for several years. Her fundi showed slight color changes of the RPE. Fine yellow dots were observed in the macula, and the retinal vessels were normal. The degeneration gradually progressed, and the retinal vessels became narrower. However, pigmentation of the retina was still sparse when she was 71 years old. Goldmann kinetic perimetry showed a large ring scotoma when she was 42 years old. She lost her residual central vision at age 55 years when her decimal visual acuity had decreased to 0.01 OD and hand motions OS (Table 1). The b-waves of the flash ERGs at age 42 years were reduced, resulting in a negative-type ERGs (Figure 5). The rod ERGs at age 66 were almost nonrecordable, but cone ERGs were reduced but recordable, i.e., rod-cone dystrophy. Her OCT images showed an absence of the outer layer of the retina in both eyes (Figure 5).

### 2.6. Transmission Electron Microscopy (TEM)

TEM was performed on the peripheral lymphocytes of *Patients 1* and *2* (Figure 6). In *Patient 1*, sparse myelin-like lamellar structures were detected in and around the nuclei and mitochondria of 40.6% (67/165) of the lymphocytes. No myelin-like lamellar structures were detected in the lysosomes. TEM of *Patient 2* found no myelin-like lamellar structures in 302 lymphocytes, except for one lymphocyte (0.3%).

## 3. Discussion

Genetic studies revealed three possible disease-causing *DRAM2* variants, viz., p.Arg236_Val237insGly, p.Arg74His, and p.Trp3del, in our three families (Figure 1, Tables S1 and S2). These were all novel mutations. In *Patients 1* and *2*, the allelic frequencies for p.Arg236_Val237insGly and p.Arg74His in the general population were extremely low, and the pathogenicity classification, according to the ACMG guideline [11], was “Likely Pathogenic” for both mutations. *Patients 1* and *2* had similar retinal degeneration with early macular involvement, and the changes were similar to those reported [5,6,7,8]. Therefore, the variants in *DRAM2* in *Patients 1* and *2* were most likely responsible for retinal degeneration. On the other hand, the pathogenicity classification of p.Trp3del, a *DRAM2* variant found in *Patient 3* in the homozygous state, was “uncertain significance” according to the ACMG guideline [11]. In addition, an *EYS* with a HAF variant, p.Gly843Glu, was also detected in a heterozygous state in *Patient 3*. However, this heterozygous *EYS* variant was not considered causative for her retinopathy because *EYS* variants have been reported to lead to *EYS*-associated retinal dystrophy only in the biallelic condition [12,13,14,15]. Therefore in *Patient 3*, the *DRAM2* variant, p.Trp3del, was considered to be responsible for her retinopathy. Overall, we concluded that the phenotypes of *Patients 1*, *2*, and *3* to be pure *DRAM2*-associsted retinopathy.

*DRAM2*-associated retinopathy has been reported as CORD21, i.e., cone-rod dystrophy. However, *Patient 1* reported night blindness before her visual acuity was reduced. The ERGs of *Patients 2* and *3* suggested a rod-cone dystrophy, which was consistent with their night blindness (Figure 4 and Figure 5). Negative flash ERGs in *Patient 3* may be related to her night blindness.

The patients with *DRAM2*-associated retinopathy reported by Abad-Morales et al. [8] complained of photophobia as an initial symptom, and their cone ERGs were reduced (cone dysfunction). On the other hand, *Patient gc17004* in Sergouniotis et al.’s report [6] had a greater reduction of the rod than the cone ERGs, i.e., rod-cone dysfunction. Another patient with *DRAM2*-associated retinopathy had dark-adaptation difficulties that started in her third decade of life (rod dysfunction) [7]. We conclude from these findings and our findings that *DRAM2*-associated retinopathy exhibits not only cone-rod dysfunction but also rod-cone dysfunction.

The clinical course of our patients suggested a relatively rapid progression (*Patient 1*) and poor visual outcome (*Patients 2* and *3*) of the retinopathy. Notably, the full-field ERGs in *Patient 1* were rapidly reduced during the 5-year follow-up (Figure 3), although her funduscopic abnormality was limited to the macular area. The FAF images in *Patient 1* at age 43 years showed slight abnormalities in the midperiphery, suggesting that the retinal degeneration was expanding to the periphery.

**Figure 7:**
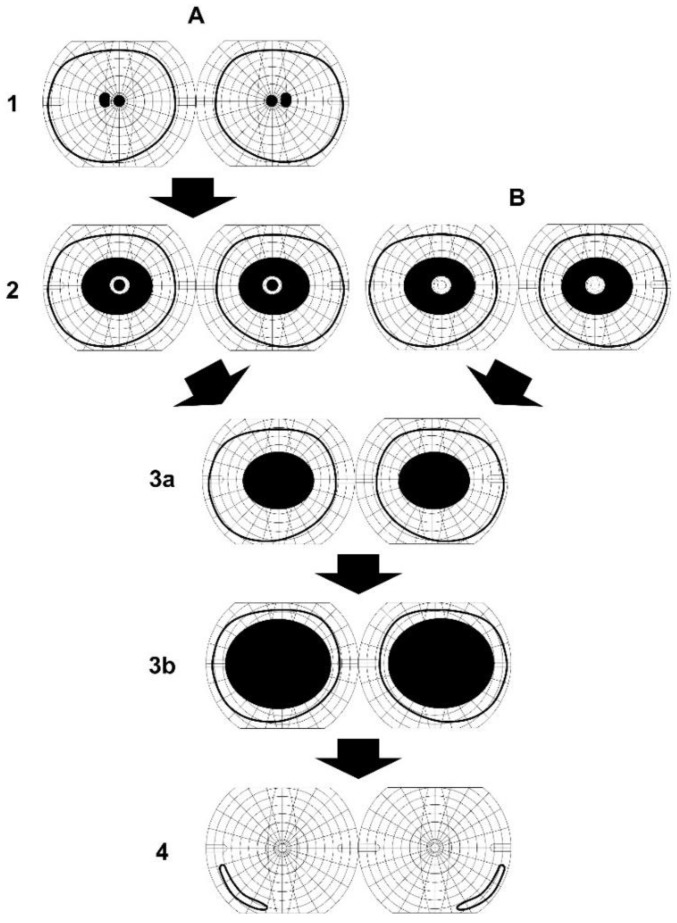
Schematic progression of the visual field defects in patients with *DRAM2*-associated retinopathy. A central scotoma appears initially (**A1**) followed by large ring scotoma (**A2**). They combine resulting in a large central scotoma (3a,3b). Ring scotoma may appear initially (**B2**). The residual central vision will be lost resulting in a large central scotoma (3a,3b). Both types A and B have poor visual outcome (4).

The findings in *Patients 2* and *3* are the longest clinical observation of 29 years among all reported patients, and the findings showed the most advanced stage of the retinopathy ever reported. Their visual outcome was poor, although both patients had no visual complaints until their third decade of life. The pattern of visual-field-defect progression may be a characteristic of *DRAM2*-associated retinopathy (Figure 4, Figure 5, and Figure 7).

*DRAM2* plays a role in autophagy, which is essential for cell survival by relieving stress through recycling or removing damaged organelles and debris by the lysosomes. In the retina, autophagy plays an important role in cellular metabolism [16] and in preventing light-induced retinal damage in photoreceptors [17]. Deficiency of the autophagy regulatory gene, *Atg7,* results in light-induced retinal degeneration [18] or RPE degeneration [19] in mice. The inhibiting function of the macroautophagy gene, *Atg5*, results in both cone [20] and rod dysfunction [21]. Hydroxychloroquine, an autophagy inhibitor, can be associated with similar retinopathy as *DRAM2*-associated retinopathy [22,23].

*DRAM2* is expressed ubiquitously in systemic organs. However, the already reported patients [5,6,7,8] and our patients had no systemic symptoms or signs except for retinal degeneration. The cause of the exclusive retinal involvement, despite the ubiquitous expression of the *DRMA2* gene, was not determined.

We investigated the effects of the *DRAM2* variants on the peripheral lymphocytes by TEM in *Patients 1* and *2*. We discovered sparse myelin-like lamellar structures in and around the nuclei and mitochondria (Figure 6). The myelin-like lamellar structures indicated an accumulation of abundant products and abnormalities of cellular metabolism [24,25]. Interestingly, the sparse myelin-like lamellar structures were observed in 40.6% of all the peripheral lymphocytes in *Patient 1,* whereas in only 0.3% in *Patient 2*. These facts suggest that the systemic effects of *DRAM2* mutations may be variable. Conversely, 60% of peripheral lymphocytes were normal in *Patient 1*, suggesting the reason why she was systemically healthy. We suggest that the DRAM protein family [1] may function complementarily when a part of them is dysfunctional in the cells.

The discrepancy between the clinical findings and lymphocyte abnormalities in *Patients 1* and *2* is unexplained. Both *Patients 1* and *2* showed typical clinical findings of *DRAM2*-associated retinopathy, although degrees of abnormalities in their peripheral lymphocytes were different (40.6% and 0.3% in *Patients 1* and *2*, respectively).

Electron microscopic findings of the photoreceptors and RPE cells have never been reported in patients with *DRAM2*-associated retinopathy. However, *DRAM2* malfunction may affect photoreceptors and RPE cells more severely than cells in other organs, possibly because of their own high-level of metabolism or lack of DRAM-family cooperation in the retina. *DRAM2* may play an important and unique role in the retina. Alternatively, patients with systemic dysfunction suffered by *DRAM2* malfunction may possibly be unable to survive until the retinopathy is expressed.

This is the only study that has examined intracellular structures in patients with *DRAM2* mutations. Further studies of the systemic effects in *DRAM2* mutations are needed to determine the systemic expressions of this mutation.

## 4. Participants and Methods

### 4.1. Ethics Statement

This was a multicenter study, and the research protocol was approved by the Ethics Review Boards of Kindai University Faculty of Medicine (22-132, approval date 2 February 2011), The Jikei University School of Medicine (24-231 6997, approval date 1 December 2012), National Institute of Sensory Organs (R11-003: approval date 6 June, 2011, and R14-050, approval date 7 July 2014), and Nippon Medical School (27-02, approval date 12 July 2016). The research protocol conformed to the tenets of the Declaration of Helsinki of the World Medical Association, and written informed consent was obtained from all patients for the examinations after the procedures had been explained in detail. All clinical data were presented after obtaining signed informed consent for their publication from the patient.

### 4.2. Genetic Studies

We have examined the WES data of 1470 patients and 840 unaffected family members from 1253 unrelated families with inherited retinal dystrophies in the database of the Japan Eye Genetics Consortium Studies (JEGC Studies) [26]. We also examined the WES data of 85 affected patients and 57 unaffected family members from 61 unrelated families with inherited retinal dystrophies in the database of Nippon Medical School Chiba Hokusoh Hospital.

Genomic DNA was extracted from all the affected subjects and unaffected family members. WES was performed with a targeted analysis of 271 retinal disease-associated genes (RetNet) [27] according to published methods [28]. The identified variants were filtered with an allelic frequency of less than 1% of the Human Genetic Variation Database (HGVD) [10] and the Japanese Multi Omics Reference Panel (jMorp) [29], which are two allele frequency databases specific for the Japanese population, and the gnomAD database [9]. The depth and coverage for the targeted areas were examined with the Integrative Genomics Viewer [30] to detect structural variants. All detected variants were analyzed with three different prediction programs; SIFT [31], PROVEAN [32], and PolyPhen-2 [33]. The pathogenicity classification of all detected variants was performed based on the guidelines of the American College of Medical Genetics and Genomics (ACMG) [11]. Together with the clinical findings of the affected subjects, the mode of inheritance in the pedigree of the disease-causing variants were determined from the called variants in the retinal disease-associated genes. All identified *DRAM2* variants were confirmed by Sanger sequencing, and each variant was compared with the NCBI Reference Sequence (NM_178454.4).

To search for other possible disease-causing variants in the families carrying the *DRAM2* variants, we re-evaluated the WES data of the patients. In brief, because a variant with HAF of more than 1% of the general population in the *EYS* gene (p.Gly843Glu) was examined in our cohorts as potentially disease-causing, we also re-examined the variants for the families. Together with phenotypic features and inheritance data, the *EYS* variant with HAF was determined.

### 4.3. Clinical Studies

We reviewed the medical records of patients with biallelic *DRAM2* variants, which were detected by the genetic studies. The results of clinical examinations included visual acuity measurements, dilated ophthalmoscopy, fluorescein fundus angiography, visual field testing, OCT imaging (Cirrus HD-OCT; Carl Zeiss Meditec AG, Dublin, CA, USA), FAF imaging (Spectralis HRA; Heidelberg Engineering, Heidelberg, Germany, California; Optos, Inc, Marlborough, MA), and the recording of the full-field ERGs with the International Society for Clinical Electrophysiology of Vision (ISCEV) standards [34] were evaluated. Details of the ERG procedures and conditions have been reported [35,36,37].

### 4.4. Transmission Electron Microscopy (TEM)

The preparations of peripheral lymphocytes for TEM were performed, as reported in detail [38]. Briefly, whole blood was centrifuged at 1000× *g* for 15 min, and the supernatant on the buffy coat was removed. The buffy coat was fixed at room temperature for 30 min by overlaying 2% glutaraldehyde in 0.1 M phosphate buffer (pH 7.3) on the buffy coat. The collected buffy coat was fixed with the same fixative solution overnight at 4 °C and then postfixed in 1% osmium tetroxide in 0.1 M phosphate buffer (pH 7.3) for 2 h at 4 °C, dehydrated in ethanol, immersed in absolute propylene oxide, and embedded in Epok 812 (Oken, Tokyo, Japan). Ultrathin sections were cut with a diamond knife, stained with uranyl acetate and lead citrate, and examined with the JEM-1400plus TEM (JEOL, Tokyo, Japan) at 100 kV.

## 5. Conclusions

In conclusion, *DRAM2*-associated retinopathy is a rare inherited retinal dystrophy in Japan. The retinopathy is associated with rod-cone dysfunction, and the prognosis is poor. The effect of the *DRAM2* variants on systemic organs may be variable.

## Figures and Tables

**Table 1:** Visual acuities and clinical courses of the *Patients.*

Patient #	Sex	Age (years)		Follow-Up Period (years)	Visual Acuity (Decimal)				Refractive Error	Others
		Initial	Final		Initial (age; years)		Intermediate (age; years)	Final (age; years)		
**1** Jikei-176-1241	F	38	43	5	Right	0.3 (38)	--	0.2 (43)	S−1.0D	Phakia No cataract
					Left	0.6 (38)	--	0.3 (43)	S−0.75D	
**2** Kinki-12-1022	M	42	71	29	Right	0.2 (42)	0.03 (55)	H.M. (71)	S−4.0D=C−1.25	IOL implantation at age 55 in both eyes
					Left	0.09 (42)	0.02 (55)	H.M. (71)	S−4.0D	
**3** Kinki-69-1159	F	42	71	29	Right	1.2 (42)	0.01 (55)	H.M. (71)	S+0.5D=C−0.25D	Nuclear cataract in both eyes
					Left	1.2 (42)	H.M. (55)	H.M. (71)	S+0.25D	

F, female; M, male; H.M., hand motions; S, sphere; C, cylinder; D, diopter; IOL, intraocular lens.

## Supplementary Materials

Supplementary Materials can be found at https://www.mdpi.com/1422-0067/21/4/1331/s1.

## Abbreviations

RPE	retinal pigment epithelium
TEM	transmission electron microscopy
WES	whole exome sequence
HAF	high allele frequency
FAF	fundus autofluorescence
OCT	optical coherence tomography
ERG	electroretinogram
ISCEV	International Society for Clinical Electrophysiology of Vision
