# Supplementary material for: Clinical Course and Electron Microscopic Findings in Lymphocytes of Patients with DRAM2-Associated Retinopathy
Source: Int J Mol Sci. 2020 Feb 16;21(4):1331. doi: 10.3390/ijms21041331 (PMC7072995; doi:10.3390/ijms21041331)
Supplement: Supplementary file 1 [file ijms-21-01331-s001.docx]

**
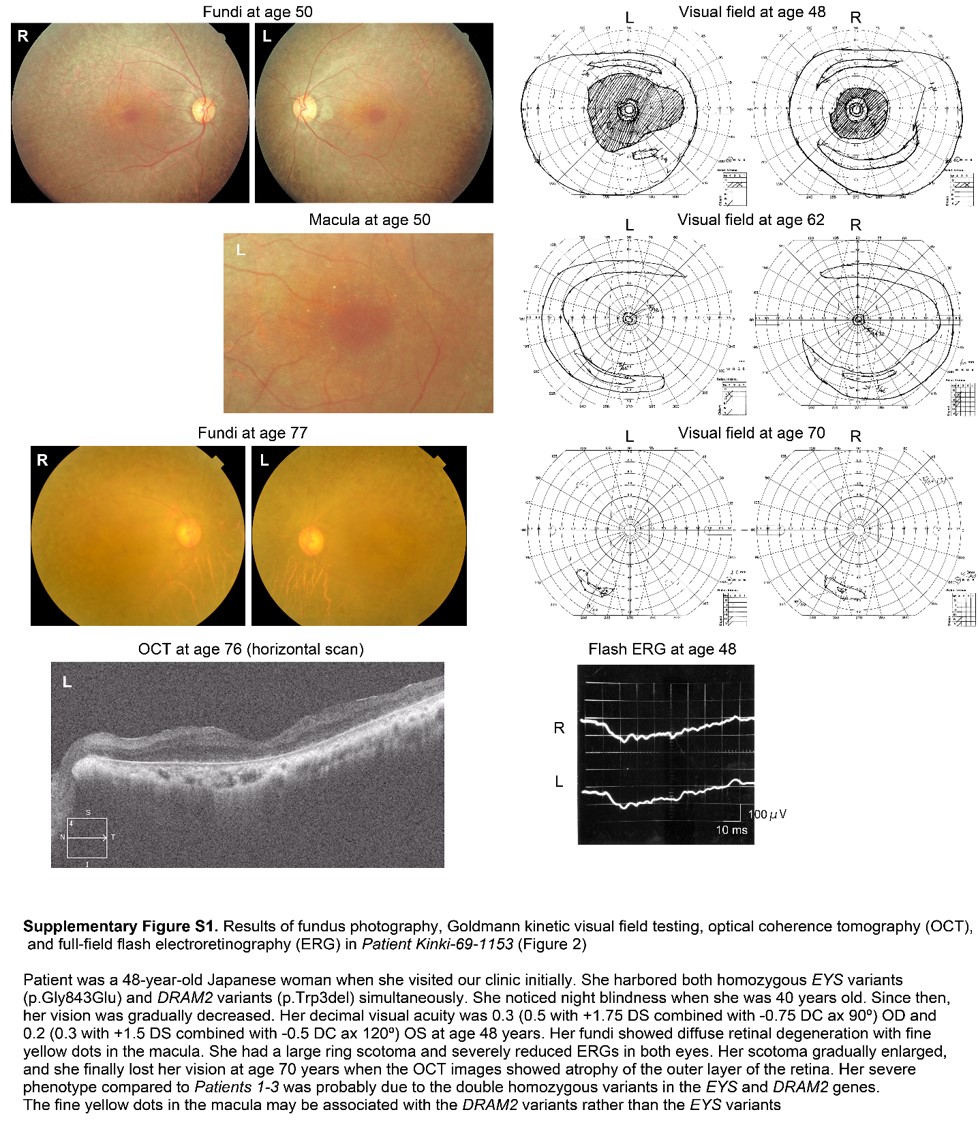
**

**Table S1.** Identification of *DRAM2* variants found in this study.

| **No.** | **Family ID** | **Consanguinity** | **HGVS.c** | **HGVS.p** | **Position** | | **Ref** | **Alt** | **Coding impact** | **Zygosity** | **Location** | **dbSNP ID** |
| --- | --- | --- | --- | --- | --- | --- | --- | --- | --- | --- | --- | --- |
|  |  |  |  |  | **Chr** | **GRCh37** |  |  |  |  |  |  |
| V1 | Jikei-176 | + | c.707-709dup | p.Arg236_Val237insGly | Chr 1 | 111,660,873 | A | ACCC | inframe insertion | Homo | Exon 9 of 9 | - |
| V2 | Kinki-12 | - | c.221G>A | p.Arg74His | Chr 1 | 111,667,482 | C | T | missense | Homo | Exon 5 of 9 | rs759094596 |
| V3 | Kinki-69 | + | c.8_10delGGT | p.Trp3del | Chr 1 | 111,674,166 | AACC | A | inframe deletion | Homo | Exon 3 of 9 | rs770539997 |

**Table S2.** Results of *in silico* allele frequency, prediction and pathogenicity analysis of identified variants in *DRAM2*

| **No.** | **HGVS.c** | **HGVS.p** | **HGVD** | **jMorp** | **gnomAD allele frequency** | | | | | **Functional prediction** | | | | | | | **ACMG Classification** | | | | | |
| --- | --- | --- | --- | --- | --- | --- | --- | --- | --- | --- | --- | --- | --- | --- | --- | --- | --- | --- | --- | --- | --- | --- |
|  |  |  |  |  | **East Asian** | **South Asian** | **African** | **European (Non-Finnish)** | **Total** | **SIFT** | | **PROVEAN** | | **Polyphen2** | | **Verdict** | | **Idntified classification rules** | | | | |
|  |  |  |  |  |  |  |  |  |  | **Prediction** | **Score** | **Prediction** | **Score** | **HDIV_pred** | **HDIV_score** |  |  |  |  |  |  |  |
| V1 | c.707-709dup | p.Arg236_Val237insGly | 0.000% | 0.010% | 0.000% | 0.000% | 0.000% | 0.000% | 0.000% | NA | NA | Deleterious | -6.61 | NA | NA | Likely Pathogenic | | PM2 | PM3 | PM4 | PP3 | PP4 |
| V2 | c.221G>A | p.Arg74His | 0.000% | 0.000% | 0.000% | 0.000% | 0.000% | 0.002% | 0.001% | Damaging | 0.261 | Deleterious | -4.64 | Probably Damaging | 1.000 | Likely Pathogenic | | PM2 | PM3 | PP3 | PP4 | - |
| V3 | c.8_10delGGT | p.Trp3del | 0.061% | 0.000% | 0.025% | 0.003% | 0.000% | 0.021% | 0.012% | NA | NA | Deleterious | -5.51 | NA | NA | Uncertain Significance | | PM4 | PP1 | PP4 |  |  |

**Table S3.** Results of *in silico* allele frequency, prediction and pathogenicity analysis of identified variants in *EYS*

| **Chr** | **Position** | **dbSNP ID** | **HGVS.c** | **HGVS.p** | **HGVD** | **jMorp** | **gnomAD allele frequency** | | | | | **Functional prediction** | | | | | | **ACMG Classification** | | | |
| --- | --- | --- | --- | --- | --- | --- | --- | --- | --- | --- | --- | --- | --- | --- | --- | --- | --- | --- | --- | --- | --- |
|  | **GRCh37** |  |  |  |  |  | **East Asian** | **South Asian** | **African** | **European (Non-Finnish)** | **Total** | **SIFT** | | **PROVEAN** | | **Polyphen2** | **Polyphen2** | **Verdict** | **Idntified classification rules** | | |
|  |  |  |  |  |  |  |  |  |  |  |  | **Prediction** | **Score** | **Prediction** | **Score** | **HDIV_pred** | **HDIV_score** |  |  |  |  |
| Chr 6 | 65,622,490 | rs74419361 | c.2528G>A | p.Gly843Glu | 2.247% | 1.71% | 0.00037% | 0.000% | 0.000% | 0.000% | 0.000026% | Damaging | 0.001 | Deleterious | -7.91 | Probably Damaging | 1.000 | Uncertain Significance | PM2 | PP3 | PP5 |
| NM_001142800.2 | |  |  |  |  |  |  |  |  |  |  |  |  |  |  |  |  |  |  |  |  |
